# Therapeutic Advances of Rare ALK Fusions in Non-Small Cell Lung Cancer

**DOI:** 10.3390/curroncol29100618

**Published:** 2022-10-16

**Authors:** Yan Xiang, Shiyu Zhang, Xiaoxu Fang, Yingying Jiang, Tingwen Fang, Jinwen Liu, Kaihua Lu

**Affiliations:** Department of Oncology, The First Affiliated Hospital of Nanjing Medical University, Nanjing 210029, China

**Keywords:** anaplastic lymphoma kinase, tyrosine kinase inhibitor, rare fusion, non-small cell lung cancer

## Abstract

Non-small cell lung cancer (NSCLC) accounts for approximately 85% of all lung cancer cases and is the leading cause of cancer-related death. Despite advances in chemotherapy and immunotherapy, the prognosis for advanced patients remains poor. The discovery of oncogenic driver mutations, such as anaplastic lymphoma kinase (ALK) mutations, means that a subset of patients has opportunities for targeted therapy. With the improvement of genetic testing coverage, more and more ALK fusion subtypes and ALK partners have been discovered, and more than 90 rare ALK fusion subtypes have been found in NSCLC. However, unlike the common fusion, echinoderm microtubule-associated protein-like 4 (EML4)-ALK, some rare ALK fusions such as striatin (STRN)-ALK and huntingtin interacting protein 1 (HIP1)-ALK, etc., the large-scale clinical data related to its efficacy are still immature. The clinical application of ALK-tyrosine kinase inhibitors (ALK-TKIs) mainly depends on the positivity of the ALK gene, regardless of the molecular characteristics of the fusion partner. Recent clinical studies in the ALK-positive NSCLC population have demonstrated differences in progression-free survival (PFS) among patients based on different ALK fusion subtypes. This article will introduce the biological characteristics of ALK fusion kinase and common detection methods of ALK fusion and focus on summarizing the differential responses of several rare ALK fusions to ALK-TKIs, and propose corresponding treatment strategies, so as to better guide the application of ALK-TKIs in rare ALK fusion population.

## 1. Introduction

Anaplastic lymphoma kinase (ALK) mutation plays an important role in the occurrence and development of non-small cell lung cancer [1,2]. The ALK gene encodes a single transmembrane receptor tyrosine kinase that belongs to the insulin receptor superfamily [3,4,5], which has an extracellular domain, a transmembrane segment, and a cytoplasmic receptor kinase segment [6]. Studies have identified pleiotrophin, midkine, and heparin as putative ALK ligands [7,8]. Usually, the ligand binds to the extracellular domain, promotes the coupling of two adjacent ALK proteins on the cell membrane, then activates the intracellular signal pathways such as RAS-MAPK, PI3K-AKT, JAK-STAT, MEKK2/3-MEK5-ERK5, CRKL-C3G and promotes cell growth [9,10].

So far, four detection methods and six ALK tyrosine kinase inhibitors (ALK-TKIs) have been approved for clinical use. As far as ALK gene detection is concerned, fluorescence in situ hybridization (FISH) is the preferred gold standard method; immuno-histochemistry (IHC) can be used for screening because it is simple and cheap, and the FDA-approved antibody (Ventana D5F3) can be used to detect ALK fusion independently without FISH validation. Reverse transcription polymerase chain reaction (RT-PCR) can also be used, but it cannot detect some rare fusions. Next-generation sequencing (NGS) can not only detect ALK fusion but also determine the type of fusion and other accompanying driver genes, which meets the current clinical needs. The emergence of ALK-TKIs has completely changed the treatment strategy and prognosis of advanced NSCLC patients with ALK fusion [11,12]. The first-generation targeted drug simultaneously has three targets: ALK, ROS1, and C-MET. The PROFILE 1014 study demonstrated that, compared with standard platinum-based chemotherapy, crizotinib significantly prolonged the median progression-free survival (mPFS, 10.9 months vs. 7.0 months; *p* < 0.001) and objective response rate (ORR, 74% vs. 45%; *p* < 0.001) of previously untreated patients with ALK-positive advanced NSCLC [13]. The second-generation ALK inhibitors can be used for crizotinib resistance such as L1196M and C1156Y mutations [14]. Lorlatinib, the third-generation targeted drug for ALK, can inhibit almost all resistance mutations that lead to crizotinib resistance, such as G1202R, G1202del, etc. [15].

Although ALK fusion is a clinically proven tumor therapeutic target, compared with other carcinogenic drivers, such as epidermal factor growth receptor (EGFR), how to better target ALK fusion lacks accuracy. More than 90 different ALK fusion partners have been identified in lung cancer, including EML4, STRN, KIF5B, HIP1, TPM-3/−4, DCTN1, GCC2, TFG, etc. [16,17,18,19,20,21,22,23]. Several clinical trials have demonstrated the efficacy of ALK-TKIs in EML4-ALK-positive NSCLC [24]. The Alex study reported that regardless of the EML4-ALK variant, the progression-free survival (PFS) of untreated ALK-positive NSCLC treated with alectinib was better than that of crizotinib [25]. However, unlike the classic EML4-ALK, some rare fusions such as STRN-ALK and HIP1-ALK, etc., the large-scale clinical data related to efficacy are still immature. The clinical application of ALK-TKIs mainly depends on the positivity of the ALK gene, regardless of the molecular characteristics of the fusion partner. Recent clinical studies in patients with ALK-positive NSCLC have shown that there are differences in PFS based on different subtypes [26]. Therefore, understanding the responses of different rare ALK fusions to ALK-TKIs is necessary to guide clinical treatment. This article will introduce the biological characteristics of ALK fusion kinases and the common detection methods of ALK fusion and focus on summarizing the rare fusions other than EML4-ALK and their treatment progress, and propose corresponding treatment strategies, so as to better guide the application of ALK-TKIs in rare ALK fusion population.

## 2. The Biology of ALK Fusion Kinases

**Figure 1:**
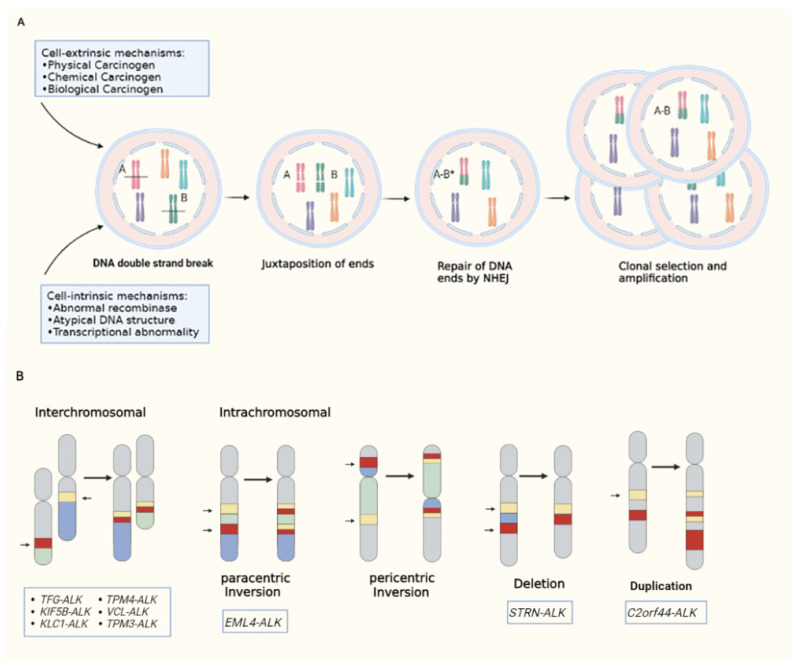
(**A**): Generation of pathogenic fusion genes; (**B**): types of ALK rearrangement.

It is generally believed that the formation of pathogenic fusion genes requires three steps (Figure 1A): firstly, external factors (such as various physical, chemical and biological factors) or internal mechanisms of cells cause DNA double-strand breaks; secondly, the ends of the broken DNA are close to each other; thirdly, the DNA ends are aberrantly repaired, probably by alternative non-homologous end-joining [27]. DNA junctions often show short homology, called microhomology, which allows non-homologous ends to be connected. In the last step, the expression of the fusion gene gives the cell growth and/or survival advantages, so as to achieve selective cloning and amplification [28].

ALK fusion is the result of chromosome rearrangement between the ALK gene and other genes (Figure 1B). One of the most common types of rearrangement is interchromosomal translocation, which involves the exchange of chromosomal material between heterologous chromosomes, such as TFG-ALK, KIF5B-ALK, etc. [29,30]. Intrachromosomal rearrangement is also usual, especially with paracentric inversion (excluding centromere). For example, EML4-ALK involves the short arm of chromosome 2 [31]. Two other intrachromosomal rearrangements are deletions and duplications, such as STRN-ALK (partial sequence deletion of the short arm of chromosome 2 results in fusion of STRN exon 3 and ALK exon 20) [32], C2orf44-ALK (caused by a 5.2-megabase pair tandem duplication on chromosome 2) [33]. However, the production of ALK fusion protein needs other conditions. Firstly, the ALK gene breakpoint must include the entire tyrosine kinase domain (usually at exon 20). Secondly, the promoter region tends to be derived from the fusion partner, probably due to the fact that the ALK promoter is inactive in adults and thus cannot drive gene transcription [34]. Finally, the fusion partner must contain the oligomerization domain. Most fusion partners contain coiled coils or leucine zipper domains that drive fusion kinase activation [1,35].

The molecular structure and biochemical properties of fusion proteins can affect the differential response of patients to ALK-TKIs, including: (1) the type of oligomeric domains; (2) the stoichiometry of oligomerization: dimers, trimers, and multimers [36,37,38,39]; (3) the intrinsic kinase activity of the fusion protein [40]; (4) protein–protein interactions, which vary mainly by the structure of the fusion partner; (5) length of the 5′ partner; (6) protein folding and spatial structure; (7) protein stability. For example, EML4-ALK variants 1, 2, and 3 have different structures. EML4-ALK variant 3 lacks the HELP and WD40 domains, while the WD40 domain of EML4-ALK variant 2 has 5 WD40 repeats less than that of variant 1, resulting in differences in protein stability and tyrosine kinase activity among the three variants [41]. In one study, Yoshida et al. [42] reported differential responses to crizotinib in 35 patients with NSCLC harboring different EML4-ALK variants. The results showed that EML4-ALK variant 1 showed better efficacy compared with non-variant 1, with the objective response rate (ORR) of 74% versus 63% (*p* = 0.7160, no significant difference), disease control rate (DCR) 95% versus 63% (*p* < 0.05, significant difference) and the PFS11 months versus 4.2 months (*p* < 0.05, significant difference).

## 3. Detection Methods for ALK Rearrangements

### 3.1. Immuno-Histochemistry

**Figure 2:**
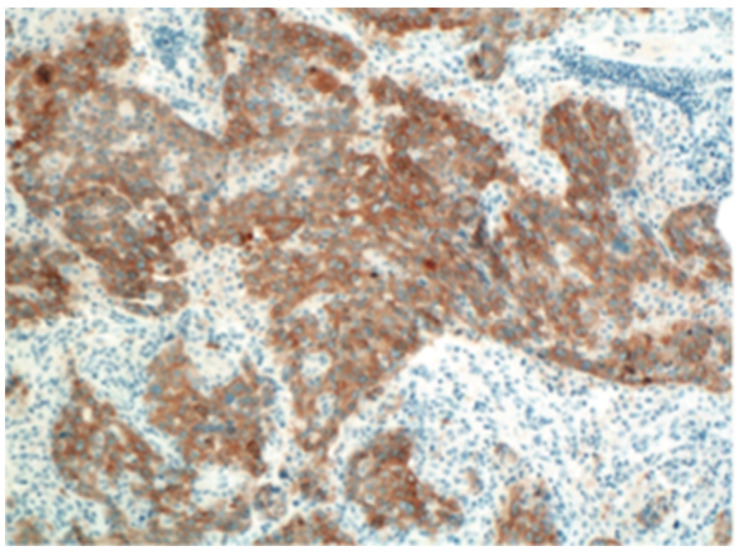
Immuno-histochemistry (IHC); From Roche Ventana.

IHC is mainly detected by the binding reaction between high-sensitivity ALK antibody and antigen, combined with signal cascade amplification (Figure 2). Normal lung tissue is difficult to express detectable levels of ALK, but the level of ALK expressed by rearranged ALK-positive NSCLC is moderate [43,44,45], and the combination of highly sensitive ALK antibodies makes IHC quite reliable in detecting ALK-positive NSCLC [43,46]. However, due to the heterogeneity of tumor cells and the uneven expression of target proteins, IHC detection may be false negative in some cases. In addition, IHC cannot identify the type and molecular structure of fusion partners, which may have an impact on treatment [26]. Therefore, clinicians recommend the use of IHC for preliminary screening [47].

### 3.2. Fluorescence in Situ Hybridization

**Figure 3:**
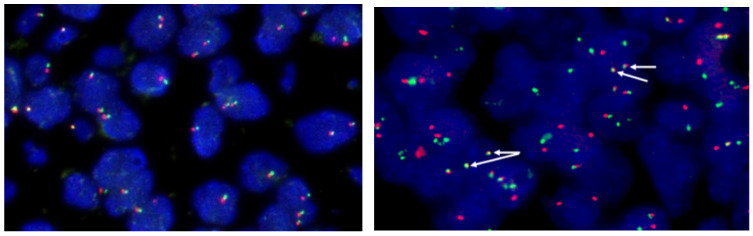
FISH interpretation criteria: (From Abbott Molecular, Vysis probes) (I) Count 50 tumor cells: If the number of positive cells was ≥50% (25), the patient was judged to have ALK rearrangements (**On the right**). If the number of positive cells was less than 10% (5), the patient was judged to have no ALK recombination (**On the left**); (II) If the positive cells were ≥10% (5) and <50% (25), another 50 tumor cells were counted, and then the 100 tumor cells were counted together. If the number of positive cells is ≥15%, the patient is judged to have ALK recombination. If the number of positive cells was less than 15%, the patient was judged to have no ALK recombination. Note: The positive cells were the cells with orange and green signal separation or orange signal alone.

FISH is to use specific nucleic acid probes labeled with fluorescence to hybridize with corresponding DNA molecules in cells, and then observe the fluorescence signal under the microscope to determine the position of DNA molecules binding to specific fluorescent probes in chromosomes [48,49] (Figure 3). FISH is a very sensitive and rapid method, which makes up for the false negative defect of IHC due to different expression intensities of ALK protein [50]. However, for some specific rearrangements, such as the separation of EML4 and ALK on chromosome 2p by only 12.5 Mb, it is impossible to accurately determine whether the two signals are separated under the microscope [51]; In addition, like IHC, FISH can only be used to determine whether the ALK site is broken, and cannot distinguish the type of fusion partner. Despite these deficiencies, FISH remains the gold standard for the detection of ALK rearrangements [52].

### 3.3. Reverse Transcription Polymerase Chain Reaction

**Figure 4:**
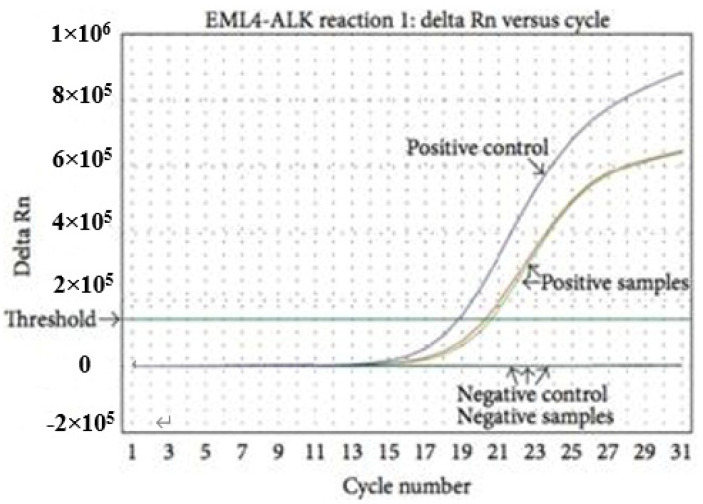
Realtime RT-PCR; From AmoyDx EML4-ALK Fusion Gene Detection Kit.

RT-PCR detects fusion mutations by extracting total RNA from tissues or cells, using the mRNA as a template, and then using pre designed primers to reverse transcribe sample RNA(Figure 4). Clinical studies have shown that different ALK fusion partners may affect the dimerization of fusion kinases, resulting in differences in tumor biological characteristics [53]. Therefore, it is important to identify specific fusion partners before selecting appropriate treatment measures. RT-PCR can sensitively detect the type of ALK fusion partner, and it is also applicable to some specimens that are not suitable for slice preparation, such as bronchial lavage fluid, pleural effusion, or blood [54]. However, the accuracy of RT-PCR diagnosis largely depends on the RNA quality of samples [54,55]. Before the successful identification of ALK fusion partners, many different primer sets need to be used, and unknown fusion variants cannot be detected [56,57].

### 3.4. Next-Generation Sequencing

**Figure 5:**
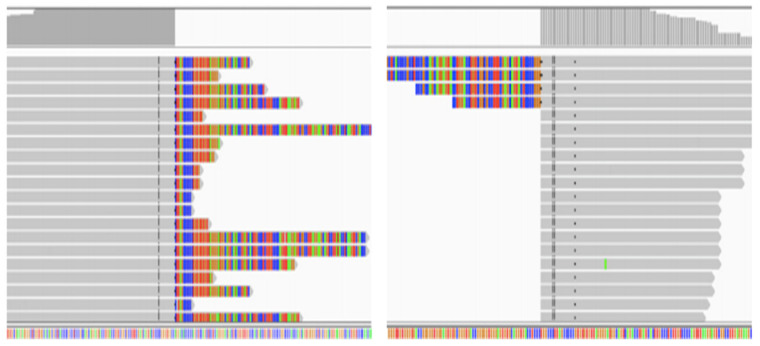
High-throughput sequencing Kit (next-generation sequencing technology, NGS).

NGS can simultaneously detect hundreds of gene mutations, insertions, deletions, fusions, copy number variations, etc., providing an effective and accurate alternative for FISH testing to detect known and new ALK fusions. Compared with traditional pathological detection, NGS has the advantages of high efficiency, high throughput and short cycle (Figure 5). As mentioned above, not all ALK fusions have biological significance. In order to become an oncogene, the fused ALK gene needs to retain its own kinase domain and the original correct reading frame. NGS can clearly observe the breaking site, so it can clearly know whether the fusion gene formed has normal biological function. In addition, NGS can also predict the therapeutic effect and drug resistance mechanism of drugs by detecting circulating tumor DNA (ctDNA) and circulating free DNA (cfDNA) in the blood, which is expected to improve the prognosis [58].

## 4. Rare ALK Fusions and Therapeutic Advances 

### 4.1. STRN-ALK

The STRN-ALK fusion in NSCLC was first described by Majewski et al. in 2013 using RNA sequencing [59]. Striatin (STRN) is a protein encoded by the STRN gene, including STRN, STRN3, and STRN4. Members of the striatin family feature multiple protein-binding domains, such as a caveolin-binding motif, a coiled-coil structure, a calmodulin-binding site, and a WD-repeat domain [60]. The STRN-ALK fusion has been reported to involve an intrachromosomal translocation of exon 3 of STRN and exon 20 of ALK within the short arm of chromosome 2 (S3A20, separated by ~7.5 Mb) [32,61,62]. STRN induces constitutive activation of ALK kinase through dimerization mediated by the 5′ coiled-coil domain in the gene [32]. Previous studies have shown that STRN-ALK can affect the aggressive characteristics of tumors, including lymph nodes, and distant metastases [32,63,64].

More than 20 cases of STRN-ALK fusion have been reported worldwide, including thyroid carcinoma, NSCLC, colorectal cancer, and renal cancer [62,65,66,67]. Among them, there were seven cases of NSCLC (Table 1). Four patients received first-line treatment with alectinib [18,64,68,69], three patients responded well to alectinib, while the other developed within 3 months [64]. Through the study of this progressive case, it was found that the patient was also accompanied by overexpression of ATP binding cassette subfamily B member 1 (ABCB1) mRNA, and this mechanism was proved to be a potential drug resistance factor of ALK-TKIs [70], so this patient responded poorly to alectinib. Li et al. reported a patient with stage IIIA ALK-positive lung adenocarcinoma who was treated with alectinib after operation [71]. After 3 months, multiple lung progression occurred. NGS showed a rare STRN-ALK fusion with MET amplification. The patient then received second-line treatment with crizotinib and achieved partial remission (PR) one month later. PFS exceeded 11 months. It has been proved that MET amplification is a recurrent drug resistance mechanism of the second-generation ALK-TKIs [15,72]. Given the high selectivity and strong affinity of alectinib for ALK fusions, crizotinib exhibits a relatively low affinity for ALK and can target multiple tyrosine kinases such as MET and ROS1 [73,74]. The progress of this patient is likely due to the occurrence of acquired MET amplification. In contrast, patients with STRN-ALK benefit more significantly from second-line or multi-line treatment with crizotinib. Zhou et al. reported a case of stage IV NSCLC patients with acquired resistance mutation of STRN-ALK after receiving osimertinib [75]. The researchers gave gefitinib combined with crizotinib in combination with gene testing results and PFS lasted for more than 6 months, which once again proved the feasibility of crizotinib in the treatment of STRN-ALK fusion.

### 4.2. KIF5B-ALK

The KIF5B gene is located on the short arm of human chromosome 10 and encodes the kinesin family 5B gene (KIF5B). The KIF5B protein is the main component of the microtubule-associated motor protein complex, which mediates the transport of organelles in eukaryotic cells [29,77]. Exons 1 to 24 of KIF5B can fuse with exon 20 of ALK to produce a new fusion gene KIF5B-ALK, which mediates ALK dimerization through the domain of KIF5B, thereby activating its tyrosine kinase activity [29]. As one of the rare fusion partners of ALK, KIF5B only accounts for about 0.4% of ALK fusions. Studies have shown that KIF5B-ALK transfected cells have significantly enhanced proliferation, migration, and invasion [16]. 

Takeuchi et al. reported a patient with lung adenocarcinoma harboring KIF5B-ALK fusion, in which intron 24 of KIF5B was fused to intron 19 of ALK [29] (Table 2). Wong et al. reported another variant of the KIF5B-ALK fusion, KIF5B exon 15 fused to ALK exon 20 [16]. However, neither case reported the sensitivity of KIF5B-ALK to ALK-TKIs. Zeng et al. introduced a case of lung adenocarcinoma with rare KIF5B-ALK (intron 20 of KIF5B is connected to intron 20 of ALK) and obtained PFS for 11 months after treatment with crizotinib [78]. In addition, NGS-ctDNA genomic analysis after craniocerebral progression in the patient suggested the known KIF5B-ALK fusion and ALK exon 23 L1196M missense mutation. The patient immediately received second-line treatment with ceritinib, craniocerebral lesions were significantly reduced and a 9-month PFS was achieved with continuous follow-up. Although the initial effect of crizotinib is remarkable, patients inevitably develop resistance. Clinical studies have shown that 66.7% of patients have secondary mutations in the ALK kinase domain after treatment with crizotinib. These drug-resistant mutations include L1196M, G1269A, G1202R and C1156Y [79,80,81,82]. Among them, L1196M is the most common secondary mutation in NSCLC. It is located in the protein kinase domain of ALK protein and can control the entry of small molecule ALK inhibitors into the hydrophobic pocket within the catalytic site, thereby sterically blocking the binding of crizotinib to ALK [83]. Second-generation ALK-TKIs are known to be effective against ALK-related secondary resistance mutations. Another preclinical evaluation showed that ceritinib can overcome crizotinib-induced resistance mutations, especially the secondary L1196M mutation [84]. Therefore, patients with KIF5B-ALK rare fusion can flexibly combine the first-, second-, and third-generation ALK-TKIs for sequential therapy in combination with ALK fusion type and mutation type, so as to improve the prognosis of patients with ALK-positive NSCLC.

### 4.3. HIP1-ALK

Huntingtin interacting protein 1 (HIP1) contains multiple domains, and its N-terminal homologous domain can bind to inositol polyphosphate signal, playing an important role in clathrin-mediated receptor transport and cell survival [85,86]. Studies have shown that HIP1 can be overexpressed in various human tumor cells and promote their clonal proliferation. Kalchman et al. reported a novel HIP1-ALK fusion gene in NSCLC for the first time [87]. The HIP1-ALK protein contains the coiled-coil domain of HIP1 and the near membrane intracellular region of ALK. Through the dimerization of the coiled-coil domain, the activity of ALK tyrosine kinase is abnormally activated, resulting in the occurrence of tumor [12,88].

A total of five HIP1-ALK variants have been reported so far: (H28:A20); (H21:A20); (H19:A20); (H22:A21) and (H30:A20) [20,24,89,90,91,92,93,94] (Table 3). Clinical studies have shown that HIP1-ALK-positive patients carrying different variants have significantly different responses to ALK-TKIs. Fang et al. tested the sensitivity to crizotinib by establishing a patient-derived xenograft (PDX) model of NSCLC with HIP1-ALK fusion (H28:A20) in vitro [93]. Unfortunately, this PDX model is derived from moderately differentiated squamous cell carcinoma, and it has not been further validated whether the patients have clinical benefits. On this basis, Couëtoux et al. successfully that lung adenocarcinoma patients with HIP1-ALK (H28:A20) responded well to crizotinib, and the PFS could reach 26.9 months [94]. In addition, Hong et al. reported a case of a postoperative patient with HIP1-ALK fusion (H21:A20) who received adjuvant crizotinib and did not experience recurrence or metastasis after 15 months of follow-up [20]. However, some studies have shown that HIP1-ALK patients with variants such as (H19:A20) and (H30:A20) have a poor response to crizotinib, in which Li, M. et al. found that HIP1-ALK (H19:A20) primary resistance to crizotinib and subsequent second-line treatment with alectinib resulted in PFS of more than 9 months [24]. Given that, Ou, S.H. et al. reported that HIP1-ALK (H30:A20) was ineffective against crizotinib [90]. Li Y et al. proved that HIP1-ALK (H30:A20) positive patients can benefit from alectinib with a PFS of more than 19 months [91]. Another retrospective study assessed clinicopathological features, genomic features, responses to ALK-TKIs, and resistance mechanisms in 11 patients with HIP1-ALK fusion from China [95]. The ORR of 10 patients treated with crizotinib was 90% [9/10 cases, 95% confidence interval (CI): 54.1%–99.5%], the mPFS was 17.9 months [95% CI: 5.8-NA], and the median overall survival (mOS) was 58.8 months (95% CI: 24.7-NA). One patient receiving first-line treatment with lorlatinib achieved partial response (PR) for more than 26.5 months, of the ten patients who received crizotinib, four underwent biopsy after progression, and two carried acquired ALK mutations (L1152V/Q1146K and L1196M). Although the HIP1-ALK fusion initially responds well to crizotinib, resistance is inevitable, whereas brigatinib is effective in patients who have failed crizotinib due to the L1152V/Q1146K resistance mutations, which may be related to the high affinity of brigatinib with these mutations.

### 4.4. Other Rare ALK Fusions

In addition to the above rare ALK fusions, there are also some fusions with very low incidence that respond well to ALK-TKIs (Table 4). Cao et al. reported a new form of ALK rearrangement (NCOA1-ALK) in which the patient received PFS for more than 18 months under the treatment of crizotinib [96]. Fang et al. introduced the first lung adenocarcinoma patient carrying myosin phosphatase interacting protein (MPIP) -ALK fusion based on RNA sequencing [97]. Previously, in lung cancer or other tumors, it has been found that MPRIP can interact with neurotrophic tyrosine receptor kinase 1 (NTRK1), platelet-derived growth factor receptorβ (PDGFRB), and proto-oncogene such as RAF1 [98,99,100]. An in vitro study found that the MPRIP-ALK fusion gene could promote colony formation, cell growth, and ALK phosphorylation. The corresponding cells were inhibited by crizotinib treatment. Consistent with the study result, the good response of MPRIP-ALK fusion to ALK-TKIs was validated in the clinical presentation of this patient, who was still receiving crizotinib at the time of reporting, with a PFS of at least 11 months. In another multi-sample retrospective study, seven patients were confirmed by NGS sequencing to carry eight rare non-EML4 variants, including CHRNA7-ALK, LOC349160-ALK, TACR1-ALK, KIF5B-ALK, CENPA-ALK, HIP1-ALK, DYSF-ALK and ITGAV-ALK [89]. The seven patients were subsequently treated with crizotinib, with five PRs, two SDs, and a median PFS of 11 months, ranging from 4 to 23 months. Recently, Chen et al. introduced a novel SOS1-ALK fusion [101]. According to related research reports, the son of sevenless homolog 1 (SOS1) gene encodes the SOS1 protein, which is a regulatory protein that is widely expressed in cells. As a key protein in the signaling pathway, SOS1 plays an important role in the regulation of many signal transduction pathways in cells, such as Ras and Rac [102,103]. Abnormal expression or mutation of SOS1 is closely related to the occurrence of clinical diseases. Through clinical observation, this SOS1-ALK fusion (S2:A20) showed a good response to crizotinib, and the patient’s PFS exceeded 6 months. A patient with novel LMO7-ALK fusion (L16:A20) reported by Li M et al. rapidly progressed within two months of first-line treatment with alectinib, and then the patient switched to the second-line treatment of ensartinib [71]. Under the preliminary follow-up, PFS has been more than 18 months. 

The patients who harbor double ALK fusion variants are extremely rare. Few investigations have focused on the concomitance of double ALK rearrangements because of the low incidence (Table 5). According to our literature search results, only eleven cases have been previously reported, including CCNY-ALK and ATIC-ALK [104], NLRC4-ALK and EML4-ALK [105], PRKCB-ALK and EML4-ALK [106], EML4-ALK and BCL11A-ALK [107], EML6-ALK and FBXO11-ALK [108], DYSF-ALK and ITGAV-ALK [109], ALK-SSH2 and EML4-ALK [110], ARID2-ALK and EML4-ALK [110], EML4-ALK and CDK15-ALK [111], PDK1-ALK and STRN-ALK [68], as well as ALK-GCA and EML4-ALK [112], etc. Previous reports confirmed that patients with double ALK fusion may respond to ALK-TKIs. However, the responses are heterogeneous for patients with different ALK fusions. The effectiveness of ALK-TKI treatment might be affected by the two kinds of ALK mutations exist simultaneously in one patient. In other words, the disappearance of one ALK fusion in patients with double ALK fusion may be the reason affecting the therapeutic effect of ALK-TKIs. Additionally, one study speculated that coexistence of double ALK fusion may be related to the occurrence of serious adverse events or drug resistance.

However, not all rare fusion types are sensitive to ALK-TKIs. Previous studies have shown that fusion partners need to provide dimerization domains to facilitate the automatic activation of kinases. Similar to other tyrosine kinases, ALK must dimerize to automatically activate and signal downstream [1]. Many fusion partners contain coiled coil domains or other known dimerization domains, but not all fusion partners have obvious dimerization motifs. PTPN3-ALK is predicted to be unresponsive to crizotinib treatment because it lacks the ALK kinase domain [113]. In addition, studies have also reported that CMTR1-ALK does not respond to crizotinib. It is hypothesized that this particular type of ALK fusion is a null fusion and thus unable to translate the kinesin that causes tumorigenesis [114].

## 5. Conclusions

Since the discovery of the EML4-ALK fusion in NSCLC, a variety of ALK-TKIs have been developed to treat ALK-positive NSCLC. The rapid development of targeted therapy has resulted in significant improvements in PFS and OS in patients with metastatic ALK-positive NSCLC. However, the heterogeneity of clinical response exists not only among different ALK fusion subtypes, but also among different variants. Among the rare fusions of ALK, we found that even in homozygous fusions, different variants responded differently to ALK-TKIs.

There are two possible explanations for the heterogeneity of responses to ALK-TKIs: one is that different fusion partners lead to differences in protein stability and expression levels, and the other is other genetic alterations that accompany ALK rearrangements leading to different responses to ALK-TKIs. In this article, we summarize that different 5’ partners affect the biological properties of ALK fusion proteins, including kinase activity, protein stability, transformation potential, and most importantly, the response to ALK-TKIs. Patients with rare ALK fusion can combine the type of ALK fusion and the resistance or sensitivity of existing mutations to different ALK-TKIs, and flexibly combine the first-, second-, and third-generation ALK inhibitors for sequential treatment to improve the prognosis.

FISH, as the gold standard for detecting ALK-positive, cannot identify specific fusion forms. Since different variants may have different responses to ALK-TKIs, it is critical to identify specific variants in different individuals to enable precision drug therapy in the future. To some extent, NGS may be a better complementary detection method. With the application and popularization of NGS technology, more and more rare ALK fusions have been discovered one after another, helping ALK-positive NSCLC patients receive more precise targeted therapy. At the same time, NGS can also detect the ctDNA and cfDNA in the blood to predict the therapeutic effect and drug resistance mechanism of the drug, thereby improving the prognosis of patients with ALK-positive NSCLC.

However, due to the limited number of rare fusion cases, it is difficult to compare the reasons for the differential responses of different rare fusions to ALK-TKIs and their resistance mechanisms. In the future, genomic, transcriptomic, and proteomic analyses are needed to investigate overall therapeutic strategies for rare fusions in ALK. At the same time, clinicians are also encouraged to report these novel fusions and provide information on fusion breakpoints and responses to ALK-TKIs to better understand the application of ALK-TKIs in rare ALK rearrangements.

## Figures and Tables

**Table 1:** Published details of STRN-ALK-positive NSCLC patients treated with ALK-TKIs.

	Ref	Year	Accompanying Mutations	ALK-TKIs	Treatment	Response
1	Yang, Y. et al. [76]	2017	MYC amplification; TP53 (R181C)	Crizotinib	Third line	CR > 6 m
2	Zhou, C. et al. [75]	2019	EGFR (19DEL)	Crizotinib +Gefitinib	Third line	PR > 6 m
3	Nakanshi, Y. et al. [64]	2017	ABCB1 mRNA overexpression	Alectinib	First line	PD < 3 m
4	Su, C. et al. [18]	2020	GRM8 (E508K); SETD2 (E1553K)	Alectinib	First line	PR > 19 m
5	Nagasaka, M. et al. [69]	2020	TP53 (L43fs);MYC amplification	Alectinib	First line	PR > 6 m
6	Zeng, H. et al. [68]	2020	PDK1-ALK (P7: A20); TP53	Alectinib	First line	PR > 7 m
7	Li, M. et al. [71]	2021	MET amplification	Alectinib Crizotinib	First line Second line	PD: 3 m PFS > 11 m

**Table 2:** Published details of KIF5B-ALK-positive NSCLC patients treated with ALK-TKIs.

	Ref	Year	Variants	ALK-TKIs	Treatment	Response
1	Takeuchi, K. et al. [29]	2009	(K24:A19)	Not treated with ALK-TKIs	–	–
2	Wong, D.W. et al. [16]	2011	(K15:A20)	Not treated with ALK-TKIs	–	–
3	Zeng, H. et al. [78]	2021	(K20:A20)	Crizotinib Ceritinib	First line Second line	PFS: 11 m PFS > 9 m

**Table 3:** Published details of HIP1-ALK-positive NSCLC patients treated with ALK-TKIs.

	Ref	Year	Variants	ALK-TKIs	Treatment	Response
1	Fang, D.D. et al. [93]	2014	(H28:A20)	Not treated with ALK-TKIs	–	PDX is sensitive to crizotinib
2	Hong, M., et al. [20]	2014	(H21:A20)	Crizotinib	First line	PFS > 15 m
3	Ou, S.H., et al. [90]	2014	(H30:A20)	Crizotinib Alectinib	First line Second line	PD PFS > 12 m
4	Jang, J. S, et al. [92]	2016	(H19:A20)	Not treated with ALK-TKIs	–	Not reported
5	Couetoux, D. T. M, et al. [94]	2019	(H28:A20)	Crizotinib	First line	PR PFS:26.9 m
6	Tian, P, et al. [89]	2020	(H22:A21)	Crizotinib	First line	PR PFS: 7.0 m
7	Li, M, et al. [24]	2021	(H19:A20)	Crizotinib Alectinib	First line Second line	PD PFS > 9 m
8	Li Y, et al. [91]	2022	(H30:A20)	Alectinib	First line	PFS > 19 m

**Table 4:** Published details of other rare ALK fusions positive NSCLC patients treated with ALK-TKIs.

	Ref	Rare ALK Fusion Types	Merge Mutations	ALK-TKIs	Treatment	Response
1	Cao Q, et al. [96]	NCOA1–ALK	CDA^K27Q^, ERCC1^N118N^, DPYD^I543V^, MTHFR^A222V^,GSTP1^I105V^	Crizotinib	Third line	PFS > 18 m
2	Fang, W. et al. [97]	MPIP-ALK		Crizotinib	Second line	PFS > 11 m
3	Tian, P. et al. [89]	CHRNA7-ALK		Crizotinib	First line	PFS: 18 m
4	Tian, P. et al. [89]	LOC349160-ALK		Crizotinib	First line	PFS: 7 m
5	Tian, P. et al. [89]	TACR1-ALK		Crizotinib	First line	PFS: 15 m
6	Tian, P. et al. [89]	CENPA-ALK		Crizotinib	First line	PFS: 4 m
7	Tian, P. et al. [89]	DYSF-ALK ITGAV-ALK	ALK p.Q1146P; MET p.M636V	Crizotinib	First line	PFS: 23 m
8	Chen, H.F. et al. [101]	SOS1-ALK		Crizotinib	First line	PFS > 6 m
9	Li, M. et al. [71]	LMO7-ALK	NRG1 c.602A > T;TP53	Alectinib Ensartinib	First line Second line	PD PFS > 18 m

**Table 5:** Published details of other double ALK fusion positive NSCLC patients treated with ALK-TKIs.

	Ref	Year	Double Fusion	ALK-TKIs	Treatment	Response
1	Wu, X. et al. [104]	2020	CCNY-ALK ATIC-ALK	Crizotinib	First line	PR > 6 m
2	Wu, X. et al. [105]	2020	NLRC4-ALK;EML4-ALK	Crizotinib	First line	PFS > 10 m
3	Luo, J. et al. [106]	2019	PRKCB-ALK; EML4-ALK	Crizotinib Ceritinib	First line Second line	PFS: 6 m PFS > 2 m
4	Qin, B. et al. [107]	2019	BCL11A-ALK; EML4-ALK	Crizotinib	First line	PFS: 13 m
5	Lin, H. et al. [108]	2018	FBXO11-ALK; EML6-ALK	Crizotinib	Second line	PFS > 11 m
6	Yin, J. et al. [109]	2018	DYSF-ALK; ITGAV-ALK	Crizotinib	Second line	PFS > 3 m
7	Tao, H. et al. [110]	2022	ALK-SSH2; EML4-ALK	Crizotinib	First line	PFS: 9 m
8	Tao, H. et al. [110]	2022	ARID2-ALK; EML4-ALK	Crizotinib	First line	PFS: 12 m
9	Guo, J. et al. [111]	2020	CDK15-ALK; EML4-ALK	Crizotinib	Second line	PFS: 23 m
10	Zeng, H. et al. [68]	2021	PDK1-ALK; STRN-ALK	Alectinib	First line	PFS > 11 m
11	Zhai, X. et al. [112]	2022	ALK-GCA; EML4-ALK	Alectinib	First line	PFS > 20 m

## References

[1] Katayama R., Lovly C.M., Shaw A.T. (2015). Therapeutic targeting of anaplastic lymphoma kinase in lung cancer: A paradigm for precision cancer medicine. Clin. Cancer Res. Off. J. Am. Assoc. Cancer Res..

[2] Ducray S.P., Natarajan K., Garland G.D., Turner S.D., Egger G. (2019). The Transcriptional Roles of ALK Fusion Proteins in Tumorigenesis. Cancers.

[3] Kong X., Pan P., Sun H., Xia H., Wang X., Li Y., Hou T. (2019). Drug Discovery Targeting Anaplastic Lymphoma Kinase (ALK). J. Med. Chem..

[4] Wolfstetter G., Pfeifer K., Backman M., Masudi T.A., Mendoza-García P., Chen S., Sonnenberg H., Sukumar S.K., Uçkun E., Varshney G.K. (2020). Identification of the Wallenda JNKKK as an Alk suppressor reveals increased competitiveness of Alk-expressing cells. Sci. Rep..

[5] Uçkun E., Wolfstetter G., Anthonydhason V., Sukumar S.K., Umapathy G., Molander L., Fuchs J., Palmer R.H. (2021). In vivo Profiling of the Alk Proximitome in the Developing Drosophila Brain. J. Mol. Biol..

[6] Du X., Shao Y., Qin H.F., Tai Y.H., Gao H.J. (2018). ALK-rearrangement in non-small-cell lung cancer (NSCLC). Thorac. Cancer.

[7] Stoica G.E., Kuo A., Powers C., Bowden E.T., Sale E.B., Riegel A.T., Wellstein A. (2002). Midkine binds to anaplastic lymphoma kinase (ALK) and acts as a growth factor for different cell types. J. Biol. Chem..

[8] Murray P.B., Lax I., Reshetnyak A., Ligon G.F., Lillquist J.S., Natoli E.J., Shi X., Folta-Stogniew E., Gunel M., Alvarado D. (2015). Heparin is an activating ligand of the orphan receptor tyrosine kinase ALK. Sci. Signal..

[9] Sampson J., Ju H.M., Song J.Y., Fry A.M., Bayliss R., Choi J. (2022). A Polytherapy Strategy Using Vincristine and ALK Inhibitors to Sensitise EML4-ALK-Positive NSCLC. Cancers.

[10] Chen S., Wang B., Fu X., Liang Y., Chai X., Ye Z., Li R., He Y., Kong G., Lian J. (2021). ALKAL1 gene silencing prevents colorectal cancer progression via suppressing Sonic Hedgehog (SHH) signaling pathway. J. Cancer.

[11] Hirsch F.R., Suda K., Wiens J., Bunn P.A. (2016). New and emerging targeted treatments in advanced non-small-cell lung cancer. Lancet (Lond. Engl.).

[12] Golding B., Luu A., Jones R., Viloria-Petit A.M. (2018). The function and therapeutic targeting of anaplastic lymphoma kinase (ALK) in non-small cell lung cancer (NSCLC). Mol. Cancer.

[13] Solomon B.J., Cappuzzo F., Felip E., Blackhall F.H., Costa D.B., Kim D.W., Nakagawa K., Wu Y.L., Mekhail T., Paolini J. (2016). Intracranial Efficacy of Crizotinib Versus Chemotherapy in Patients With Advanced ALK-Positive Non-Small-Cell Lung Cancer: Results From PROFILE 1014. J. Clin. Oncol. Off. J. Am. Soc. Clin. Oncol..

[14] Yanagitani N., Uchibori K., Koike S., Tsukahara M., Kitazono S., Yoshizawa T., Horiike A., Ohyanagi F., Tambo Y., Nishikawa S. (2020). Drug resistance mechanisms in Japanese anaplastic lymphoma kinase-positive non-small cell lung cancer and the clinical responses based on the resistant mechanisms. Cancer Sci..

[15] Gainor J.F., Dardaei L., Yoda S., Friboulet L., Leshchiner I., Katayama R., Dagogo-Jack I., Gadgeel S., Schultz K., Singh M. (2016). Molecular Mechanisms of Resistance to First- and Second-Generation ALK Inhibitors in ALK-Rearranged Lung Cancer. Cancer Discov..

[16] Wong D.W., Leung E.L., Wong S.K., Tin V.P., Sihoe A.D., Cheng L.C., Au J.S., Chung L.P., Wong M.P. (2011). A novel KIF5B-ALK variant in nonsmall cell lung cancer. Cancer.

[17] Vendrell J.A., Taviaux S., Béganton B., Godreuil S., Audran P., Grand D., Clermont E., Serre I., Szablewski V., Coopman P. (2017). Detection of known and novel ALK fusion transcripts in lung cancer patients using next-generation sequencing approaches. Sci. Rep..

[18] Su C., Jiang Y., Jiang W., Wang H., Liu S., Shao Y., Zhao W., Ning R., Yu Q. (2020). STRN-ALK Fusion in Lung Adenocarcinoma with Excellent Response Upon Alectinib Treatment: A Case Report and Literature Review. OncoTargets Ther..

[19] Qin J., Zeng D., Xie F., Yu R., Wu X., Liu K., Shao Y.W., Lu H., Jiang J. (2019). Rare GCC2-ALK fusion G13:A20 detected by next generation sequencing in non-small cell lung cancer patients and treatment response. Transl. Cancer Res..

[20] Hong M., Kim R.N., Song J.Y., Choi S.J., Oh E., Lira M.E., Mao M., Takeuchi K., Han J., Kim J. (2014). HIP1-ALK, a novel fusion protein identified in lung adenocarcinoma. J. Thorac. Oncol..

[21] Evangelista A.F., Zanon M.F., Carloni A.C., de Paula F.E., Morini M.A., Ferreira-Neto M., Soares I.C., Miziara J.E., de Marchi P., Scapulatempo-Neto C. (2017). Detection of ALK fusion transcripts in FFPE lung cancer samples by NanoString technology. BMC Pulm. Med..

[22] Zhao S., Liu W., Li S., Shi T., Chen Q., Li Q., Sun L., Ren D., Song Z., Huang C. (2021). A Case of Simultaneously Diagnosed Lung Adenocarcinoma and Endobronchial Inflammatory Myofibroblastic Tumor with Two Distinct Types of ALK Translocation. Cancer Res. Treat..

[23] Camidge D.R., Kim H.R., Ahn M.J., Yang J.C.H., Han J.Y., Hochmair M.J., Lee K.H., Delmonte A., Garcia Campelo M.R., Kim D.W. (2021). Brigatinib Versus Crizotinib in ALK Inhibitor-Naive Advanced ALK-Positive NSCLC: Final Results of Phase 3 ALTA-1L Trial. J. Thorac. Oncol..

[24] Li M., Tang Q., Chen S., Wang Y. (2021). A novel HIP1-ALK fusion variant in lung adenocarcinoma showing resistance to Crizotinib. Lung Cancer.

[25] Camidge D.R., Dziadziuszko R., Peters S., Mok T., Noe J., Nowicka M., Gadgeel S.M., Cheema P., Pavlakis N., de Marinis F. (2019). Updated Efficacy and Safety Data and Impact of the EML4-ALK Fusion Variant on the Efficacy of Alectinib in Untreated ALK-Positive Advanced Non-Small Cell Lung Cancer in the Global Phase III ALEX Study. J. Thorac. Oncol..

[26] Li W., Guo L., Liu Y., Dong L., Yang L., Chen L., Liu K., Shao Y., Ying J. (2021). Potential Unreliability of Uncommon ALK, ROS1, and RET Genomic Breakpoints in Predicting the Efficacy of Targeted Therapy in NSCLC. J. Thorac. Oncol..

[27] Hastings P.J., Lupski J.R., Rosenberg S.M., Ira G. (2009). Mechanisms of change in gene copy number. Nat. Rev. Genet..

[28] Bunting S.F., Nussenzweig A. (2013). End-joining, translocations and cancer. Nat. Rev. Cancer.

[29] Takeuchi K., Choi Y.L., Togashi Y., Soda M., Hatano S., Inamura K., Takada S., Ueno T., Yamashita Y., Satoh Y. (2009). KIF5B-ALK, a novel fusion oncokinase identified by an immunohistochemistry-based diagnostic system for ALK-positive lung cancer. Clin. Cancer Res. Off. J. Am. Assoc. Cancer Res..

[30] Kemps P.G., Picarsic J., Durham B.H., Hélias-Rodzewicz Z., Hiemcke-Jiwa L., van den Bos C., van de Wetering M.D., van Noesel C.J.M., van Laar J.A.M., Verdijk R.M. (2022). ALK-positive histiocytosis: A new clinicopathologic spectrum highlighting neurologic involvement and responses to ALK inhibition. Blood.

[31] Maddalo D., Manchado E., Concepcion C.P., Bonetti C., Vidigal J.A., Han Y.C., Ogrodowski P., Crippa A., Rekhtman N., de Stanchina E. (2014). In vivo engineering of oncogenic chromosomal rearrangements with the CRISPR/Cas9 system. Nature.

[32] Kelly L.M., Barila G., Liu P., Evdokimova V.N., Trivedi S., Panebianco F., Gandhi M., Carty S.E., Hodak S.P., Luo J. (2014). Identification of the transforming STRN-ALK fusion as a potential therapeutic target in the aggressive forms of thyroid cancer. Proc. Natl. Acad. Sci. USA.

[33] Lipson D., Capelletti M., Yelensky R., Otto G., Parker A., Jarosz M., Curran J.A., Balasubramanian S., Bloom T., Brennan K.W. (2012). Identification of new ALK and RET gene fusions from colorectal and lung cancer biopsies. Nat. Med..

[34] Shaw A.T., Hsu P.P., Awad M.M., Engelman J.A. (2013). Tyrosine kinase gene rearrangements in epithelial malignancies. Nat. Rev. Cancer.

[35] Mizuta H., Okada K., Araki M., Adachi J., Takemoto A., Kutkowska J., Maruyama K., Yanagitani N., Oh-Hara T., Watanabe K. (2021). Gilteritinib overcomes lorlatinib resistance in ALK-rearranged cancer. Nat. Commun..

[36] Richards M.W., O’Regan L., Roth D., Montgomery J.M., Straube A., Fry A.M., Bayliss R. (2015). Microtubule association of EML proteins and the EML4-ALK variant 3 oncoprotein require an N-terminal trimerization domain. Biochem. J..

[37] Medves S., Noël L.A., Montano-Almendras C.P., Albu R.I., Schoemans H., Constantinescu S.N., Demoulin J.B. (2011). Multiple oligomerization domains of KANK1-PDGFRβ are required for JAK2-independent hematopoietic cell proliferation and signaling via STAT5 and ERK. Haematologica.

[38] Medves S., Demoulin J.B. (2012). Tyrosine kinase gene fusions in cancer: Translating mechanisms into targeted therapies. J. Cell. Mol. Med..

[39] Hirai N., Sasaki T., Okumura S., Minami Y., Chiba S., Ohsaki Y. (2020). Monomerization of ALK Fusion Proteins as a Therapeutic Strategy in ALK-Rearranged Non-small Cell Lung Cancers. Front. Oncol..

[40] Wiesner T., Lee W., Obenauf A.C., Ran L., Murali R., Zhang Q.F., Wong E.W., Hu W., Scott S.N., Shah R.H. (2015). Alternative transcription initiation leads to expression of a novel ALK isoform in cancer. Nature.

[41] Heuckmann J.M., Balke-Want H., Malchers F., Peifer M., Sos M.L., Koker M., Meder L., Lovly C.M., Heukamp L.C., Pao W. (2012). Differential protein stability and ALK inhibitor sensitivity of EML4-ALK fusion variants. Clin. Cancer Res. Off. J. Am. Assoc. Cancer Res..

[42] Yoshida T., Oya Y., Tanaka K., Shimizu J., Horio Y., Kuroda H., Sakao Y., Hida T., Yatabe Y. (2016). Differential Crizotinib Response Duration Among ALK Fusion Variants in ALK-Positive Non-Small-Cell Lung Cancer. J. Clin. Oncol. Off. J. Am. Soc. Clin. Oncol..

[43] Mino-Kenudson M., Chirieac L.R., Law K., Hornick J.L., Lindeman N., Mark E.J., Cohen D.W., Johnson B.E., Jänne P.A., Iafrate A.J. (2010). A novel, highly sensitive antibody allows for the routine detection of ALK-rearranged lung adenocarcinomas by standard immunohistochemistry. Clin. Cancer Res. Off. J. Am. Assoc. Cancer Res..

[44] Niu X., Chuang J.C., Berry G.J., Wakelee H.A. (2017). Anaplastic Lymphoma Kinase Testing: IHC vs. FISH vs. NGS. Curr. Treat. Options Oncol..

[45] Chang W.C., Kim H.K., Shin B.K. (2020). Clinicopathological features and diagnostic methods of ALK fusion-positive non-small cell lung cancer in Korea. Oncol. Rep..

[46] Park H.S., Lee J.K., Kim D.W., Kulig K., Kim T.M., Lee S.H., Jeon Y.K., Chung D.H., Heo D.S. (2012). Immunohistochemical screening for anaplastic lymphoma kinase (ALK) rearrangement in advanced non-small cell lung cancer patients. Lung Cancer.

[47] Cutz J.C., Craddock K.J., Torlakovic E., Brandao G., Carter R.F., Bigras G., Deschenes J., Izevbaye I., Xu Z., Greer W. (2014). Canadian anaplastic lymphoma kinase study: A model for multicenter standardization and optimization of ALK testing in lung cancer. J. Thorac. Oncol..

[48] Tang Z., Wang L., Tang G., Medeiros L.J. (2019). Fluorescence in Situ Hybridization (FISH) for Detecting Anaplastic Lymphoma Kinase (ALK) Rearrangement in Lung Cancer: Clinically Relevant Technical Aspects. Int. J. Mol. Sci..

[49] Lim S.M., Chang H., Cha Y.J., Liang S., Tai Y.C., Li G., Pestova E., Policht F., Perez T., Soo R.A. (2017). Validation of ALK/ROS1 Dual Break Apart FISH Probe probe in non-small-cell lung cancer. Lung Cancer.

[50] Teixidó C., Karachaliou N., Peg V., Gimenez-Capitan A., Rosell R. (2014). Concordance of IHC, FISH and RT-PCR for EML4-ALK rearrangements. Transl. Lung Cancer Res..

[51] Lazzari C., Spitaleri G., Catania C., Barberis M., Noberasco C., Santarpia M., Delmonte A., Toffalorio F., Conforti F., De Pas T.M. (2014). Targeting ALK in patients with advanced non small cell lung cancer: Biology, diagnostic and therapeutic options. Crit. Rev. Oncol. Hematol..

[52] Hout D.R., Schweitzer B.L., Lawrence K., Morris S.W., Tucker T., Mazzola R., Skelton R., McMahon F., Handshoe J., Lesperance M. (2017). Performance of a RT-PCR Assay in Comparison to FISH and Immunohistochemistry for the Detection of ALK in Non-Small Cell Lung Cancer. Cancers.

[53] Hallberg B., Palmer R.H. (2013). Mechanistic insight into ALK receptor tyrosine kinase in human cancer biology. Nat. Rev. Cancer.

[54] Karachaliou N., Rosell R. (2013). Optimal detection of ALK rearranged lung adenocarcinomas. J. Thorac. Oncol..

[55] Yu K., Xing J., Zhang J., Zhao R., Zhang Y., Zhao L. (2017). Effect of multiple cycles of freeze-thawing on the RNA quality of lung cancer tissues. Cell Tissue Bank..

[56] Murakami Y., Mitsudomi T., Yatabe Y. (2012). A Screening Method for the ALK Fusion Gene in NSCLC. Front. Oncol..

[57] Heeke S., Benzaquen J., Vallee A., Allegra M., Mazieres J., Fayada J., Rajamani J., Lee M., Ordinario E., Tiotiu A. (2021). Detection of ALK fusion transcripts in plasma of non-small cell lung cancer patients using a novel RT-PCR based assay. Ann. Transl. Med..

[58] Fukui T., Tachihara M., Nagano T., Kobayashi K. (2022). Review of Therapeutic Strategies for Anaplastic Lymphoma Kinase-Rearranged Non-Small Cell Lung Cancer. Cancers.

[59] Majewski I.J., Mittempergher L., Davidson N.M., Bosma A., Willems S.M., Horlings H.M., de Rink I., Greger L., Hooijer G.K., Peters D. (2013). Identification of recurrent FGFR3 fusion genes in lung cancer through kinome-centred RNA sequencing. J. Pathol..

[60] Du Q.Y., Yao J.H., Zhou Y.C., Xu L.J., Zhao F.Y., Yang Y. (2020). High STRN Expression Promotes HCC Invasion and Migration but Not Cell Proliferation or Apoptosis through Facilitating Epithelial-Mesenchymal Transition. BioMed Res. Int..

[61] Pérot G., Soubeyran I., Ribeiro A., Bonhomme B., Savagner F., Boutet-Bouzamondo N., Hostein I., Bonichon F., Godbert Y., Chibon F. (2014). Identification of a recurrent STRN/ALK fusion in thyroid carcinomas. PLoS ONE.

[62] Kusano H., Togashi Y., Akiba J., Moriya F., Baba K., Matsuzaki N., Yuba Y., Shiraishi Y., Kanamaru H., Kuroda N. (2016). Two Cases of Renal Cell Carcinoma Harboring a Novel STRN-ALK Fusion Gene. Am. J. Surg. Pathol..

[63] Yakirevich E., Resnick M.B., Mangray S., Wheeler M., Jackson C.L., Lombardo K.A., Lee J., Kim K.M., Gill A.J., Wang K. (2016). Oncogenic ALK Fusion in Rare and Aggressive Subtype of Colorectal Adenocarcinoma as a Potential Therapeutic Target. Clin. Cancer Res. Off. J. Am. Assoc. Cancer Res..

[64] Nakanishi Y., Masuda S., Iida Y., Takahashi N., Hashimoto S. (2017). Case Report of Non-Small Cell Lung Cancer with STRN-ALK Translocation: A Nonresponder to Alectinib. J. Thorac. Oncol..

[65] Zeng Q., Gao H., Zhang L., Qin S., Gu Y., Chen Q. (2021). Coexistence of a secondary STRN-ALK, EML4-ALK double-fusion variant in a lung adenocarcinoma patient with EGFR mutation: A case report. Anti-Cancer Drugs.

[66] Panebianco F., Nikitski A.V., Nikiforova M.N., Kaya C., Yip L., Condello V., Wald A.I., Nikiforov Y.E., Chiosea S.I. (2019). Characterization of thyroid cancer driven by known and novel ALK fusions. Endocr. Relat. Cancer.

[67] Lasota J., Chłopek M., Wasąg B., Kowalik A., Christiansen J., Lamoureux J., Kuźniacka A., Felisiak-Gołąbek A., Liu Y., Reyes T.A.R. (2020). Colorectal Adenocarcinomas Harboring ALK Fusion Genes: A Clinicopathologic and Molecular Genetic Study of 12 Cases and Review of the Literature. Am. J. Surg. Pathol..

[68] Zeng H., Li Y., Wang Y., Huang M., Zhang Y., Tian P., Li W. (2021). Case Report: Identification of Two Rare Fusions, PDK1-ALK and STRN-ALK, That Coexist in a Lung Adenocarcinoma Patient and the Response to Alectinib. Front. Oncol..

[69] Nagasaka M., Sarvadevabatla N., Iwata S., Ge Y., Sukari A., Klosowski C., Yanagihara R. (2021). STRN-ALK, A Novel In-Frame Fusion With Response to Alectinib. JTO Clin. Res. Rep..

[70] Katayama R., Sakashita T., Yanagitani N., Ninomiya H., Horiike A., Friboulet L., Gainor J.F., Motoi N., Dobashi A., Sakata S. (2016). P-glycoprotein Mediates Ceritinib Resistance in Anaplastic Lymphoma Kinase-rearranged Non-small Cell Lung Cancer. EBioMedicine.

[71] Li M., An Z., Tang Q., Ma Y., Yan J., Chen S., Wang Y. (2021). Mixed responses to first-line alectinib in non-small cell lung cancer patients with rare ALK gene fusions: A case series and literature review. J. Cell. Mol. Med..

[72] Dagogo-Jack I., Yoda S., Lennerz J.K., Langenbucher A., Lin J.J., Rooney M.M., Prutisto-Chang K., Oh A., Adams N.A., Yeap B.Y. (2020). MET Alterations Are a Recurring and Actionable Resistance Mechanism in ALK-Positive Lung Cancer. Clin. Cancer Res. Off. J. Am. Assoc. Cancer Res..

[73] Krawczyk P., Grenda A., Terlecka P., Błach J., Wojas-Krawczyk K., Kucharczyk T., Chmielewska I., Kieszko R., Jarosz B., Gil M. (2021). Crizotinib efficacy in advanced non-squamous NSCLC patients with ALK or ROS1 rearrangement. Sci. Rep..

[74] Camidge D.R., Otterson G.A., Clark J.W., Ignatius Ou S.H., Weiss J., Ades S., Shapiro G.I., Socinski M.A., Murphy D.A., Conte U. (2021). Crizotinib in Patients With MET-Amplified NSCLC. J. Thorac. Oncol..

[75] Zhou C., Zeng L., Zhang Y., Yang N. (2019). Responder of Gefitinib Plus Crizotinib in Osimertinib Failure EGFR-mutant NSCLC-Resistant With Newly Identified STRN-ALK by Next-Generation Sequencing. J. Thorac. Oncol..

[76] Yang Y., Qin S.K., Zhu J., Wang R., Li Y.M., Xie Z.Y., Wu Q. (2017). A Rare STRN-ALK Fusion in Lung Adenocarcinoma Identified Using Next-Generation Sequencing-Based Circulating Tumor DNA Profiling Exhibits Excellent Response to Crizotinib. Mayo Clin. Proc. Innov. Qual. Outcomes.

[77] Serra-Marques A., Martin M., Katrukha E.A., Grigoriev I., Peeters C.A., Liu Q., Hooikaas P.J., Yao Y., Solianova V., Smal I. (2020). Concerted action of kinesins KIF5B and KIF13B promotes efficient secretory vesicle transport to microtubule plus ends. eLife.

[78] Zeng H., Liu Y., Wang W., Tang Y., Tian P., Li W. (2021). A rare KIF5B-ALK fusion variant in a lung adenocarcinoma patient who responded to crizotinib and acquired the ALK L1196M mutation after resistance: A case report. Ann. Palliat. Med..

[79] Lin Y.T., Chiang C.L., Hung J.Y., Lee M.H., Su W.C., Wu S.Y., Wei Y.F., Lee K.Y., Tseng Y.H., Su J. (2021). Resistance profiles of anaplastic lymphoma kinase tyrosine kinase inhibitors in advanced non-small-cell lung cancer: A multicenter study using targeted next-generation sequencing. Eur. J. Cancer.

[80] Kwon M., Ku B.M., Olsen S., Park S., Lefterova M., Odegaard J., Jung H.A., Sun J.M., Lee S.H., Ahn J.S. (2022). Longitudinal monitoring by next-generation sequencing of plasma cell-free DNA in ALK rearranged NSCLC patients treated with ALK tyrosine kinase inhibitors. Cancer Med..

[81] Fontana D., Ceccon M., Gambacorti-Passerini C., Mologni L. (2015). Activity of second-generation ALK inhibitors against crizotinib-resistant mutants in an NPM-ALK model compared to EML4-ALK. Cancer Med..

[82] Bordi P., Tiseo M., Rofi E., Petrini I., Restante G., Danesi R., Del Re M. (2017). Detection of ALK and KRAS Mutations in Circulating Tumor DNA of Patients With Advanced ALK-Positive NSCLC With Disease Progression During Crizotinib Treatment. Clin. Lung Cancer.

[83] Chen J., Wang J., Zhu W. (2017). Mutation L1196M-induced conformational changes and the drug resistant mechanism of anaplastic lymphoma kinase studied by free energy perturbation and umbrella sampling. Phys. Chem. Chem. Phys. PCCP.

[84] Song Z., Wang M., Zhang A. (2015). Alectinib: A novel second generation anaplastic lymphoma kinase (ALK) inhibitor for overcoming clinically-acquired resistance. Acta Pharm. Sinica. B.

[85] Gottfried I., Ehrlich M., Ashery U. (2010). The Sla2p/HIP1/HIP1R family: Similar structure, similar function in endocytosis?. Biochem. Soc. Trans..

[86] Hsu C.Y., Lin C.H., Jan Y.H., Su C.Y., Yao Y.C., Cheng H.C., Hsu T.I., Wang P.S., Su W.P., Yang C.J. (2016). Huntingtin-Interacting Protein-1 Is an Early-Stage Prognostic Biomarker of Lung Adenocarcinoma and Suppresses Metastasis via Akt-mediated Epithelial-Mesenchymal Transition. Am. J. Respir. Crit. Care Med..

[87] Kalchman M.A., Koide H.B., McCutcheon K., Graham R.K., Nichol K., Nishiyama K., Kazemi-Esfarjani P., Lynn F.C., Wellington C., Metzler M. (1997). HIP1, a human homologue of S. cerevisiae Sla2p, interacts with membrane-associated huntingtin in the brain. Nat. Genet..

[88] Mano H. (2008). Non-solid oncogenes in solid tumors: EML4-ALK fusion genes in lung cancer. Cancer Sci..

[89] Tian P., Liu Y., Zeng H., Tang Y., Lizaso A., Ye J., Shao L., Li Y. (2020). Unique molecular features and clinical outcomes in young patients with non-small cell lung cancer harboring ALK fusion genes. J. Cancer Res. Clin. Oncol..

[90] Ou S.H., Klempner S.J., Greenbowe J.R., Azada M., Schrock A.B., Ali S.M., Ross J.S., Stephens P.J., Miller V.A. (2014). Identification of a novel HIP1-ALK fusion variant in Non-Small-Cell Lung Cancer (NSCLC) and discovery of ALK I1171 (I1171N/S) mutations in two ALK-rearranged NSCLC patients with resistance to Alectinib. J. Thorac. Oncol..

[91] Li Y., Duan P., Guan Y., Chen Q., Grenda A., Christopoulos P., Denis M.G., Guo Q. (2022). High efficacy of alectinib in a patient with advanced lung adenocarcinoma with 2 rare ALK fusion sites: A case report. Transl. Lung Cancer Res..

[92] Jang J.S., Wang X., Vedell P.T., Wen J., Zhang J., Ellison D.W., Evans J.M., Johnson S.H., Yang P., Sukov W.R. (2016). Custom Gene Capture and Next-Generation Sequencing to Resolve Discordant ALK Status by FISH and IHC in Lung Adenocarcinoma. J. Thorac. Oncol..

[93] Fang D.D., Zhang B., Gu Q., Lira M., Xu Q., Sun H., Qian M., Sheng W., Ozeck M., Wang Z. (2014). HIP1-ALK, a novel ALK fusion variant that responds to crizotinib. J. Thorac. Oncol..

[94] Couëtoux du Tertre M., Marques M., Tremblay L., Bouchard N., Diaconescu R., Blais N., Couture C., Pelsser V., Wang H., Higenell V. (2019). Analysis of the Genomic Landscape in ALK+ NSCLC Patients Identifies Novel Aberrations Associated with Clinical Outcomes. Mol. Cancer Ther..

[95] Kang J., Deng Q.M., Peng K.C., Li P., Zhu B.T., Wang P., Chu X.P., Zhong W.Z., Chen H.J., Wang W.X. (2022). Clinicopathological features and resistance mechanisms in HIP1-ALK-rearranged lung cancer: A multicenter study. Genes Chromosom. Cancer.

[96] Cao Q., Liu Z., Huang Y., Qi C., Yin X. (2019). NCOA1-ALK: A novel ALK rearrangement in one lung adenocarcinoma patient responding to crizotinib treatment. OncoTargets Ther..

[97] Fang W., Gan J., Hong S., Lu F., Zhang L. (2019). MPRIP-ALK, a Novel ALK Rearrangement That Responds to ALK Inhibition in NSCLC. J. Thorac. Oncol..

[98] Vaishnavi A., Capelletti M., Le A.T., Kako S., Butaney M., Ercan D., Mahale S., Davies K.D., Aisner D.L., Pilling A.B. (2013). Oncogenic and drug-sensitive NTRK1 rearrangements in lung cancer. Nat. Med..

[99] Stransky N., Cerami E., Schalm S., Kim J.L., Lengauer C. (2014). The landscape of kinase fusions in cancer. Nat. Commun..

[100] Naumann N., Schwaab J., Metzgeroth G., Jawhar M., Haferlach C., Göhring G., Schlegelberger B., Dietz C.T., Schnittger S., Lotfi S. (2015). Fusion of PDGFRB to MPRIP, CPSF6, and GOLGB1 in three patients with eosinophilia-associated myeloproliferative neoplasms. Genes Chromosom. Cancer.

[101] Chen H.F., Wang W.X., Xu C.W., Huang L.C., Li X.F., Lan G., Zhai Z.Q., Zhu Y.C., Du K.Q., Lei L. (2020). A novel SOS1-ALK fusion variant in a patient with metastatic lung adenocarcinoma and a remarkable response to crizotinib. Lung Cancer.

[102] Hillig R.C., Sautier B., Schroeder J., Moosmayer D., Hilpmann A., Stegmann C.M., Werbeck N.D., Briem H., Boemer U., Weiske J. (2019). Discovery of potent SOS1 inhibitors that block RAS activation via disruption of the RAS-SOS1 interaction. Proc. Natl. Acad. Sci. USA.

[103] Gerboth S., Frittoli E., Palamidessi A., Baltanas F.C., Salek M., Rappsilber J., Giuliani C., Troglio F., Rolland Y., Pruneri G. (2018). Phosphorylation of SOS1 on tyrosine 1196 promotes its RAC GEF activity and contributes to BCR-ABL leukemogenesis. Leukemia.

[104] Wu X., Zhou H., He Z., Zhang Z., Feng W., Zhao J., Chen H., Wang S., Wang W., Wang Q. (2020). Coexistence of a novel CCNY-ALK and ATIC-ALK double-fusion in one patient with ALK-positive NSCLC and response to crizotinib: A case report. Transl. Lung Cancer Res..

[105] Wu X., Wang W., Zou B., Li Y., Yang X., Liu N., Ma Q., Zhang X., Wang Y., Li D. (2020). Novel NLRC4-ALK and EML4-ALK double fusion mutations in a lung adenocarcinoma patient: A case report. Thorac. Cancer.

[106] Luo J., Gu D., Lu H., Liu S., Kong J. (2019). Coexistence of a Novel PRKCB-ALK, EML4-ALK Double-Fusion in a Lung Adenocarcinoma Patient and Response to Crizotinib. J. Thorac. Oncol..

[107] Qin B.D., Jiao X.D., Liu K., Wu Y., Zang Y.S. (2019). Identification of a Novel EML4-ALK, BCL11A-ALK Double-Fusion Variant in Lung Adenocarcinoma Using Next-Generation Sequencing and Response to Crizotinib. J. Thorac. Oncol..

[108] Lin H., Ren G., Liang X. (2018). A Novel EML6-ALK FBXO11-ALK Double Fusion Variant in Lung Adenocarcinoma and Response to Crizotinib. J. Thorac. Oncol..

[109] Yin J., Zhang Y., Zhang Y., Peng F., Lu Y. (2018). Reporting on Two Novel Fusions, DYSF-ALK and ITGAV-ALK, Coexisting in One Patient with Adenocarcinoma of Lung, Sensitive to Crizotinib. J. Thorac. Oncol..

[110] Tao H., Liu Z., Mu J., Gai F., Huang Z., Shi L. (2022). Concomitant novel ALK-SSH2, EML4-ALK and ARID2-ALK, EML4-ALK double-fusion variants and confer sensitivity to crizotinib in two lung adenocarcinoma patients, respectively. Diagn. Pathol..

[111] Guo J., Shi J., Yao M., Jin Y., Liu D., Liu W., Wang K., Jiang D. (2020). A rare double ALK fusion variant EML4-ALK and CDK15-ALK in lung adenocarcinoma and response to crizotinib: A case report. Medicine.

[112] Zhai X., Wu Q., Pu D., Yin L., Wang W., Zhu D., Xu F. (2021). Case Report: A Novel Non-Reciprocal ALK Fusion: ALK-GCA and EML4-ALK Were Identified in Lung Adenocarcinoma, Which May Respond to Alectinib Adjuvant-Targeted Therapy. Front. Oncol..

[113] Ou S.H., Bartlett C.H., Mino-Kenudson M., Cui J., Iafrate A.J. (2012). Crizotinib for the treatment of ALK-rearranged non-small cell lung cancer: A success story to usher in the second decade of molecular targeted therapy in oncology. Oncol..

[114] Du X., Shao Y., Gao H., Zhang X., Zhang H., Ban Y., Qin H., Tai Y. (2018). CMTR1-ALK: An ALK fusion in a patient with no response to ALK inhibitor crizotinib. Cancer Biol. Ther..

